# An Adaptive Homeostatic Algorithm for the Unsupervised Learning of Visual Features

**DOI:** 10.3390/vision3030047

**Published:** 2019-09-16

**Authors:** Laurent U. Perrinet

**Affiliations:** INT, Inst Neurosci Timone, Aix Marseille Univ, CNRS, 27, Bd. Jean Moulin, CEDEX 5, 13385 Marseille, France; laurent.perrinet@univ-amu.fr

**Keywords:** vision, sparseness, computer vision, unsupervised learning, neuroscience

## Abstract

The formation of structure in the visual system, that is, of the connections between cells within neural populations, is by and large an unsupervised learning process. In the primary visual cortex of mammals, for example, one can observe during development the formation of cells selective to localized, oriented features, which results in the development of a representation in area V1 of images’ edges. This can be modeled using a sparse Hebbian learning algorithms which alternate a coding step to encode the information with a learning step to find the proper encoder. A major difficulty of such algorithms is the joint problem of finding a good representation while knowing immature encoders, and to learn good encoders with a nonoptimal representation. To solve this problem, this work introduces a new regulation process between learning and coding which is motivated by the homeostasis processes observed in biology. Such an optimal homeostasis rule is implemented by including an adaptation mechanism based on nonlinear functions that balance the antagonistic processes that occur at the coding and learning time scales. It is compatible with a neuromimetic architecture and allows for a more efficient emergence of localized filters sensitive to orientation. In addition, this homeostasis rule is simplified by implementing a simple heuristic on the probability of activation of neurons. Compared to the optimal homeostasis rule, numerical simulations show that this heuristic allows to implement a faster unsupervised learning algorithm while retaining much of its effectiveness. These results demonstrate the potential application of such a strategy in machine learning and this is illustrated by showing the effect of homeostasis in the emergence of edge-like filters for a convolutional neural network.

## 1. Introduction: Reconciling Competition and Cooperation

The architecture of the visual system implements a complex dynamic system that operates at different time scales. One of its properties is to succeed in representing information quickly, while optimizing this encoding in the long-term. Respectively, these correspond to the coding and learning time scales. In the case of the mammalian primary visual cortex (V1) for instance, the results of Hubel & Wiesel [1] show that cells of V1 have predominantly relatively localized receptive fields which are selective at different orientations. As such, this rapid coding of the retinal image, of the order of 50 ms in humans, transforms the raw visual information into a rough “sketch” that represents the outlines of objects in the image by using elementary edge-like features. An important aspect of this internal representation is that it is “sparse”: for most natural images, only a relatively small number of features (also called atoms) are necessary to describe the input [2]. Thus, the coding step consists in choosing the right encoder that selects as few features as possible among a collection of them (called the dictionary). Amazingly, Olshausen & Field [3] show that when enforcing a sparse prior on the encoding step, such edge-like filters are obtained using a simple Hebbian unsupervised learning strategy.

Additionally, recent advances in machine learning, and especially on unsupervised learning, have shed new light on the functioning of the underlying biological neural processes. By definition, unsupervised learning aims at learning the best dictionary to represent the input image autonomously, that is, without using other external knowledge, such as in supervised or reinforcement learning. Algorithms that include such a process as the input to classical, supervised deep-learning show great success in tasks like image denoising [4] or classification [5,6]. A variant consists of forcing the generated representation to be sparsely encoded [7], whether by adding a penalty term to the optimized cost function or by encoding each intermediate representation by a pursuit algorithm [8]. Interestingly, [8] proposes a model of Convolutional Sparse Coding (CSC) tightly connected with a Convolutional Neural Network (CNN), so much that the forward pass of the CNN is equivalent to a CSC with a thresholding pursuit algorithm. These unsupervised algorithms are equivalent to a gradient descent optimization over an informational-type coding cost [9]. This cost makes it then possible to quantitatively evaluate the joint exploration of new learning or coding strategies. As such, this remark shows us that unsupervised learning consists of two antagonistic mechanisms, a long time scale that corresponds to the learning and exploration of new components and a faster scale that corresponds to coding, and that both are interdependent.

**Figure 1:**
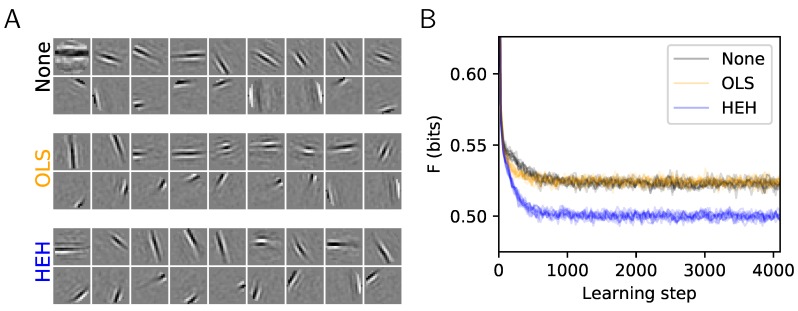
Role of homeostasis in learning sparse representations. This plot shows the results of the same Sparse Hebbian Learning algorithm at convergence (4096 learning epochs), but using different homeostasis algorithms. The compared algorithms are: None (using a simple normalization of the atoms), OLS (the method of the work by the authors of [10]), and HEH (using the optimal homeostasis rule described in this paper). (**A**) For each algorithm, 18 atoms from the N=676 filters are shown. These are of the same size as the image patches (M=21×21=441, circularly masked) and presented in each matrix (separated by a white border). The upper and lower row respectively show the least and most probably selected atoms. This highlights qualitatively the fact that without proper homeostasis, dictionary learning leads to inhomogeneous representations. (**B**) Evolution of cost *F* (in bits, see Equation (6)) as a function of the number of iterations and cross-validated over 10 runs. Whereas OLS provides a similar convergence than None, the HEH method provides quantitatively a better final convergence.

However, when exploring such algorithms, this convergence may fail to reach a global optimum. In particular, we identified that in simulations for which we aim at comparing the model with the biological substrate, such as when the number of neurons increases, the convergence gradually degenerated (see Figure 1A, “None”). An aspect often ignored in this type of learning is the set of homeostasis processes that control the average activity of neurons within a population. Indeed, there is an intrinsic complexity in unsupervised dictionary learning algorithms. On the one side, neurons are selected by the Sparse Hebbian Learning algorithm by selecting those with maximal activity. This implements a competition within neurons in a population for selecting the one which best matches the visual input. On the other hand, as the learning reinforces the match between the neuron’s response and the visual feature, a regulation process is necessary to avoid the case where only one neuron learns and the other neurons are never selected. Indeed, in such a case, the selection of this neuron would be certain and the surprise associated to this representation would be null. Such homeostatic process thus implements a form of cooperation which aims at optimizing the competition across neurons. But how to adapt the regularization parameter of each atom to make sure no atoms are wasted because of improper regularization settings?

In the original SparseNet algorithm of sparse unsupervised learning [10], homeostasis is implemented as a heuristic that prevents the average energy of each coefficient from diverging. In the majority of present unsupervised learning algorithms, it takes the form of a normalization, that is, an equalization of the energy of each atom in the dictionary [11]. In general, the neural mechanisms of homeostasis are at work in many components of the neural code and are essential to the overall transduction of neural information. For example, the subnetworks of glutamate and GABA-type neurons may regulate the overall activity of neural populations [12]. Such mechanisms could be tuned to balance the contribution of the excitatory populations with respect to that of inhibitory populations. As a consequence, this creates a so-called balanced network, which may explain many facets of the properties of the primary visual cortex [13], such as criticality and scale invariant processing of information in cortical networks, including adaptation. Such a balance may be important to properly represent distributions of activities within a population. This has been demonstrated to be beneficial for image categorization [6]. At the modeling level, these mechanisms are often implemented in the form of normalization rules [14], which are considered as the basis of a normative theory to explain the function of the primary visual cortex [15]. However, when extending such models using unsupervised learning, most effort is focused in showing that the cells’ selectivity has the same characteristics than those observed in neurophysiology [16,17,18]. Other algorithms use nonlinearities that implicitly implement homeostatic rules in neuromimetic algorithms [19] or spiking neurons [20]. These nonlinearities are mainly used in the output of successive layers of deep learning networks that are nowadays widely used for image classification or artificial intelligence. However, most of these nonlinear normalization rules are based on heuristics mimicking neural mechanisms but are not justified as part of the global problem underlying unsupervised learning. Framing this problem in a probabilistic framework allows to consider in addition to coding and learning the intermediate time scale of homeostasis and allows to associate it to an adaptation mechanism [21]. Our main argument is that, compared to classical [10] or Deep Learning approaches, including an homeostatic process optimizes unsupervised learning at both the coding and learning time scales and allows for the implementation of fast algorithms compatible with the performance of biological networks.

In this paper, we will first define a simple algorithm for controlling the selection of coefficients in sparse coding algorithms based on a set of nonlinear functions similar to generic neural gain normalization mechanisms. Such functions will be used to implement a homeostasis mechanism based on histogram equalization by progressively adapting these nonlinear functions. This algorithm will extend an already existing algorithm of unsupervised sparse learning [22] to a more general setting. We will show quantitative results of this optimal algorithm by applying it to different pairs of coding and learning algorithms. Second, we will propose a simplification of this homeostasis algorithm based on the activation probability of each neuron, thanks to the control of the slope of its corresponding Rectifying Linear Unit (ReLU). We show that it yields similar quantitative results as the full homeostasis algorithm and that it converges more rapidly than classical methods [10,23]. We designed our computational architecture to be able to quantitatively cross-validate for every single hyperparameter. All these scripts are available as open-sourced code, including the Supplementary Material. Finally, we will conclude by showing an application of such an adaptive algorithm to CNNs and discuss on its development in real-world architectures.

## 2. Unsupervised Learning and the Optimal Representation of Images

Visual items composing natural images are often sparse, such that knowing a model for the generation of images, the brain may use this property to represent images using only a few of these items. Images are represented in a matrix $y={\left(y_{k}\right)}_{k=1}^{K}\in R^{K\times M}$ as a batch of *K* vectorial samples (herein, we will use a batch size of K=256), where each image is raveled along $M=21^{2}=441$ pixels. We use image patches drawn from large images of outdoor scenes, as provided in the “kodakdb” database which is available in the project’s repository. These are circularly masked to avoid artifacts (see Annex (https://spikeai.github.io/HULK/#Loading-a-database)). Each $y_{k,j}\in R$ is the corresponding luminance value. In the context of the representation of natural images, let us assume the generic Generative Linear Model, such that for any sample *k* the image was generated as $y_{k}=\Phi^{T}a_{k}+\epsilon$, where by definition, the *N* coefficients are denoted by $a_{k}={\left(a_{k,i}\right)}_{i=1}^{N}\in R^{N}$ and the dictionary by $\Phi \in R^{N\times M}$. Finally, $\epsilon \in R^{M}$ is a Gaussian iid noise, which is normal without loss of generality by scaling the norm of the dictionary’s rows. By understanding this model, unsupervised learning aims at finding the least surprising causes (the parameters ${\hat{a}}_{k}$ and Φ) for the data $y_{k}$. In particular, the cost may be formalized in probabilistic terms as [10]

(1) $$\begin{array}{cc} F & \approx {\langle -\log\left[p\left(y_{k}|{\hat{a}}_{k},\Phi \right)p\left({\hat{a}}_{k}\right)\right]\rangle }_{k=1\ldots K} \end{array}$$

(2) $$\begin{array}{cc} \; & =\langle \frac{1}{2}\left|\;\right|y_{k}-\Phi {\hat{a}}_{k}{\left|\;\right|}_{2}^{2}-\log p\left({\hat{a}}_{k}\right)\rangle_{k=1\ldots K} \end{array}$$

Such hypothesis allows us to define, in all generality, the different costs that are optimized in most existing models of unsupervised learning. Explicitly, the representation is optimized by minimizing a cost defined on prior assumptions on representation’s sparseness, that is on $\log p({\hat{a}}_{k})$. For instance, learning is accomplished in SparseNet [10] by defining a sparse prior probability distribution function for each coefficients in the factorial form $\log p\left(a_{k}\right)∼-\beta \sum_{i}\log\left(1+\frac{a_{i}^{2}}{\sigma^{2}}\right)$, where β corresponds to the steepness of the prior and σ to its scaling (see Figure 13.2 from the work by the authors of [24]). Then, knowing this sparse solution, learning is defined as slowly changing the dictionary using Hebbian learning. Indeed, to compute the partial derivative of *F* with respect to Φ, we have simply:

(3) $$\begin{array}{cc} \frac{\partial }{\partial \Phi }F & ={\langle \frac{1}{2}\frac{\partial }{\partial \Phi_{i}}\left[{\left(y_{k}-\Phi^{T}{\hat{a}}_{k}\right)}^{T}\left(y_{k}-\Phi^{T}{\hat{a}}_{k}\right)\right]\rangle }_{k=1\ldots K} \end{array}$$

(4) $$\begin{array}{cc} \; & ={\langle {\hat{a}}_{k}\left(y_{k}-\Phi^{T}{\hat{a}}_{k}\right)\rangle }_{k=1\ldots K}. \end{array}$$

This allows to define unsupervised learning as the (stochastic) gradient descent using this equation. Similarly to Equation (17) in the work by the authors of [10] or to Equation (2) in the work by the authors of [25], the relation is a linear “Hebbian” rule [26], as it enhances the weight of neurons proportionally to the activity (coefficients) between pre- and postsynaptic neurons. Note that there is no learning for nonactivated coefficients (for which ${\hat{a}}_{k}=0$). Implementing a stochastic gradient descent, we can also use a (classical) scheduling of the learning rate and a proper initialization of the weights (see Annex (https://spikeai.github.io/HULK/#Testing-two-different-dictionary-initalization-strategies)). The only novelty of this formulation compared to other linear Hebbian learning rules, such as those in the work by the authors of [27], is to take advantage of the sparse (nonlinear) representation, hence the name Sparse Hebbian Learning (SHL). In general, the parameterization of the prior in Equation (2) has major impacts on results of the sparse coding, and thus on the emergence of edge-like receptive fields and requires proper tuning. For instance, a L2-norm penalty term (that is, a Gaussian prior on the coefficients) corresponds to Tikhonov regularization [28] and a L1-norm term (that is, an exponential prior for the coefficients) corresponds to the LASSO convex cost which may be optimized by least-angle regression (LARS) [29] or FISTA [30].

### 2.1. Algorithm: Sparse Coding with a Control Mechanism for the Selection of Atoms

Concerning the choice of a proper prior distribution, the spiking nature of neural information demonstrates that the transition from an inactive to an active state is far more significant at the coding time scale than smooth changes of the firing rate. This is, for instance, perfectly illustrated by the binary nature of the neural code in the auditory cortex of rats [31]. Binary codes also emerge as optimal neural codes for rapid signal transmission [32]. This is also relevant for neuromorphic systems which transmit discrete, asynchronous events such as a network packet or an Address-Event Representation [33]. With a binary event-based code, the cost is only incremented when a new neuron gets active, regardless to its (analog) value. Stating that an active neuron carries a bounded amount of information of λ bits, an upper bound for the representation cost of neural activity on the receiver end is proportional to the count of active neurons, that is, to the $\ell_{0}$ pseudo-norm $\left|\;\right|a_{k}{\left|\;\right|}_{0}=\left|\left\{i,a_{k,i}\neq 0\right\}\right|$:

(5) $$\begin{array}{c} F\approx \langle \frac{1}{2}\left|\;\right|y_{k}-\Phi a_{k}{\left|\;\right|}_{2}^{2}+\lambda |\;|a_{k}{\left|\;\right|}_{0}\rangle_{k=1\ldots K} \end{array}$$

This cost is similar with information criteria such as the Akaike Information Criteria [34] or distortion rate ([35] p. 571). For $\lambda =\log_{2}N$, it gives the total information (in bits) to code for the residual (using entropic coding) and the list of spikes’ addresses, as would be sufficient when using a rank-order quantization [36]. In general, the high interconnectivity of neurons (on average of the order of 10,000 synapses per neurons) justifies such an informational perspective with respect to the analog quantization of information in the point-to-point transfer of information between neurons. However, Equation (5) defines a nonconvex cost which is harder to optimize (in comparison to convex formulations in Equation (2) for instance) since the $\ell_{0}$ pseudo-norm sparseness leads to a nonconvex optimization problem, which is “NP-complete” with respect to the dimension *M* of the dictionary ([35] p. 418).

**Figure 2:**
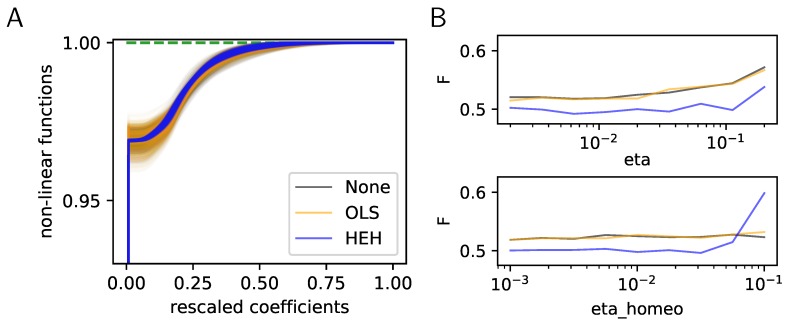
Histogram Equalization Homeostasis and its role in unsupervised learning. (**A**) Nonlinear homeostatic functions $z_{i},\forall i$ learned using Hebbian learning. These functions were computed for different homeostatic strategies (None, OLS or HEH) but only used in HEH. Note that for our choice of $N_{0}=21$ and $N=26^{2}=676$, all cumulative functions start around $1-N_{0}/N\approx 0.968$. At convergence of HEH, the probability of choosing any filter is equiprobable, while the distribution of coefficients is more variable for None and OLS. As a consequence, the distortion between the distributions of sparse coefficients is minimal for HEH, a property which is essential for the optimal representation of signals in distributed networks such as the brain. (**B**) Effect of learning rate η (eta) and homeostatic learning rate $\eta_{h}$ (eta_homeo) on the final cost as computed for the same learning algorithms but with different homeostatic strategies (None, OLS or HEH). Parameters were explored around a default value and over a 4 octaves logarithmic scale. This shows that HEH is robust across a wide range of parameters.

Still, there are many solutions to this optimization problem and here, we will use a generalized version of the Matching Pursuit (MP) algorithm ([35] p. 422), see Algorithm 1. A crucial aspect of this algorithm is the arg max function as it produces at each step a competition among *N* neurons (that is, $\log_{2}N$ bits per event). For this reason, we will introduce a mechanism to tune this competition. For any signal $y_{k}$ drawn from the database, we get the coefficients $a_{k}=S\left(y_{k};\Psi =\left\{\Phi ,z,N_{0}\right\}\right)$ thanks to the sparse coding step. The parameter $N_{0}\overset{def.}{=}\left|\;\right|a_{k}{\left|\;\right|}_{0}$ controls the amount of sparsity that we impose to the coding. The novelty of this generalization of MP lies in the scalar functions $z={\left\{z_{i}\right\}}_{i=1\ldots N}$ which control the competition for the best match across atoms. Although the absolute value function is chosen in the original MP algorithm (that is, $\forall i,z_{i}\left(a_{k}\right)=\left|a_{k}\right|$), we will define these at a first attempt as the rescaled nonlinear rectified linear unit (ReLU) with gain $\gamma_{i}$: $\forall i,z_{i}\left(a_{k,i}\right)=\gamma_{i}*a_{k,i}*\delta \left(a_{k,i}>0\right)$ where δ is Kronecker’s indicator function. We found, as in the work by the authors of [17], that by using an algorithm like Matching Pursuit (that is using the symmetric function or setting $\forall i,\gamma_{i}=1$ as in [11] for instance), the Sparse Hebbian Learning algorithm could provide results similar to SparseNet, leading to the emergence of Gabor-like edge detectors as is observed in simple cells of the primary visual cortex [37]. One advantage compared to [10] is the nonparametric assumption on the prior based on this more generic $\ell_{0}$ pseudo-norm sparseness. Importantly for our study, we observed that this class of algorithms could lead to solutions corresponding to a local minimum of the full objective function: Some solutions seem as efficient as others for representing the signal but do not represent edge-like features homogeneously (Figure 1A, None). Moreover, using other sparse coding algorithms which are implemented in the sklearn library, we compared the convergence of the learning with different sparse coding algorithms. In particular, we compared the learning as implemented with matching pursuit to that with orthogonal matching pursuit (OMP) [38], LARS or FISTA (see Supplementary Material). For all these sparse coding algorithms, during the early learning step, some cells may learn “faster” than others. These cells have more peaked distributions of their activity and tend to be selected more often (as shown in Figure 1A “None” and quantified in the variability of their distributions in Figure 2A “None”). It is thus necessary to include a homeostasis process that will ensure the convergence of the learning. The goal of this work is to study the specific role of homeostasis in learning sparse representations and to propose a homeostasis mechanism based on the functions $z_{i}$, which optimizes the learning of an efficient representation.

**Algorithm 1** Generalized Matching Pursuit: $a_{k}=S\left(y_{k};\Psi =\left\{\Phi ,z,N_{0}\right\}\right)$
1:set the sparse vector $a_{k}$ to zero,2:initialize ${\bar{a}}_{k,i}=\langle y_{k},\Phi_{i}\rangle$ for all *i*3:**while**$\left|\;\right|a_{k}{\left|\;\right|}_{0}<N_{0}$**do**:4: select the best match: $i^{*}={\mathrm{error}}_{i}\left[z_{i}\left({\bar{a}}_{k,i}\right)\right]$5: update the sparse coefficient: $a_{k,i^{*}}=a_{k,i^{*}}+{\bar{a}}_{k,i^{*}}$,6: update residual: $\forall i,{\bar{a}}_{k,i}\leftarrow {\bar{a}}_{k,i}-a_{k,i^{*}}\langle \Phi_{i^{*}},\Phi_{i}\rangle$.


### 2.2. Algorithm: Histogram Equalization Homeostasis

Knowing a dictionary and a sparse coding algorithm, we may transform any data sample $y_{k}$ into a set of sparse coefficients using the above algorithm: $a_{k}=S\left(y_{k};\Psi =\left\{\Phi ,z,N_{0}\right\}\right)$. However, at any step during learning, dictionaries may not have learned homogeneously and may as a result exhibit different distributions for the coefficients. Regrettably, this would not be taken into account in the original cost (see Equation (5)) as we assumed by hypothesis and as in [10] that the components of the sparse vector are identically distributed. To overcome this problem, we may use an additional component to the cost which measures the deviation to this hypothesis:

(6) $$F\approx \langle \frac{1}{2}\left|\;\right|y_{k}-\Phi a_{k}{\left|\;\right|}_{2}^{2}+\lambda |\;|a_{k}{\left|\;\right|}_{0}+\mu W\left(a_{k}\right)\rangle_{k=1\ldots K}$$

where we define the distance $W(a_{k})$ as the sum of the distances of each individual coefficient’s cumulative probability distribution (that we denote as $P^{i}$) to the average cumulative probability distribution $P^{0}=\frac{1}{N}\sum_{i}P^{i}$. Each distance for each atom of index *i* is defined as the earth mover’s distance (Wasserstein metric with p=1), such that $W\left(a_{k}\right)=\sum_{i}\int_{a\geq 0}\left|P^{i}\left(a\right)-P^{0}\left(a\right)\right|da$ [39]. In general, such a distance gives a measure of the solution to the well-known transportation problem between two histograms. In our setting, given a proper value for μ, this gives a lower bound of the estimate of the quantization error. Indeed, as information is coded in the address of neurons (using λ bits per coefficient) based on the average distribution of coefficients across neurons, quantization error is lowest when the activity within the neural population is uniformly balanced, that is when each coefficient value is a priori selected with the same probability. When this hypothesis does not hold, we need to transform the value of a coefficient from that which was expected (that is, the average across neurons). It can be shown that this error is proportional to the additional information (in bits) which is necessary to code the vector of coefficients compared to the case where distributions are identically distributed. In particular, a necessary and sufficient condition for minimizing this additional term is that the prior probability of selecting coefficients are identical $\forall \left(i,j\right),p\left(a_{k,i}\right)=p\left(a_{k,j}\right)$. This would result in $\forall i,P^{i}=P^{0}$ and thus $W(a_{k})=0$ and cancel the additional term. To reach this optimum, we may use different transformation functions $z_{i}$ to influence the choice of coefficients such that we may use these functions to optimize the objective cost defined by Equation (6).

To achieve this uniformity, we may define a homeostatic gain control mechanism based on histogram equalization, that is, by transforming coefficients in terms of quantiles by setting $\forall i,z_{i}\left(a\right)=P^{i}\left(a\right)\overset{def.}{=}Pr\left(a>a_{i}\right)$. Such a transform is similar to the inverse transform sampling which is used to optimize representation in auto-encoders [40] and can be considered as a nonparametric extension of the “reparameterization trick” used in variational auto-encoders [9]. Moreover, it has been found that such an adaptation mechanism is observed in the response of the retina to various contrast distributions [41]. However, an important point to note is that this joint optimization problem between coding and homeostasis is circular as we can not access the true posterior Pr(a): Indeed, the coefficients depend on nonlinear coefficients through $a_{k}=S\left(y_{k};\Psi =\left\{\Phi ,z_{i},N_{0}\right\}\right)$, whereas the nonlinear functions depend on the (cumulative) distribution of the coefficients. We will make the assumption that such a problem can be solved iteratively by slowly learning the nonlinear functions. Starting with an initial set of nonlinear functions as in None, we will derive an approximation for the sparse coefficients. Then, the function $z_{i}$ for each coefficient of the sparse vector is calculated using an iterative moving average scheme (parameterized by time constant $1/\eta_{h}$) to smooth its evolution during learning. At the coding level, this nonlinear function is incorporated in the matching step of the matching pursuit algorithm (see Algorithm 1), to modulate the choice of the most probable as that corresponding to the maximal quantile: $i^{*}={\arg\;\max}_{i}z_{i}\left(a_{i}\right)$. We will coin this variant as Histogram Equalization Homeostasis (HEH). The rest of this Sparse Hebbian Learning algorithm is left unchanged. As we adapt the dictionaries progressively during Sparse Hebbian Learning, we may incorporate this HEH homeostasis during learning by choosing an appropriate learning rate $\eta_{h}$. To recapitulate the different choices we made from the learning to the coding and the homeostasis, the unsupervised learning can be summarized using the following steps.

We compared qualitatively the set Φ of receptive filters generated with different homeostasis algorithms (see Figure 1A). A more quantitative study of the coding is shown by comparing the decrease of the cost as a function of the iteration step (see Figure 1B). This demonstrate that forcing the learning activity to be uniformly spread among all receptive fields results in a faster convergence of the representation error as represented by the decrease of the cost *F*.

### 2.3. Results: A More Efficient Unsupervised Learning Using Homeostasis

We have shown above that we can find an exact solution to the problem of homeostasis during Sparse Hebbian Learning. However, this solution has several drawbacks. First, it is computationally-intensive on a conventional computer as it necessitates to store each $z_{i}$ function to store the cumulative distribution of each coefficient. More importantly, it seems that biological neurons seem to rather use a simple gain control mechanism. This can be implemented by modifying the gain $\gamma_{i}$ of the slope of the ReLU function to operate a gradient descent on the cost based on the distribution of each coefficients. Such strategy can be included in the SHL algorithm by replacing line 9 in the learning algorithm (see Algorithm 2) by $z_{i}\left(a\right)=\gamma_{i}\;\cdot \;a\;\cdot \;\delta \left(\;\cdot \;>0\right)$. For instance, the strategy in SparseNet [10] assumes a cost on the difference between the observed variance of coefficients $V_{i}$ as computed over a set of samples compared to a desired value $\sigma_{g}^{2}$ (and assuming a multiplicative noise parameterized by α) :

(7) $$\begin{array}{c} V_{i}\leftarrow \left(1-\eta_{h}\right)\;\cdot \;V_{i}+\eta_{h}\;\cdot \;1/K\sum_{k=1\cdots K}a_{i,k}^{2} \end{array}$$

(8) $$\begin{array}{cc} \; & \mathrm{and}\gamma_{i}\leftarrow \gamma_{i}\;\cdot \;{\left(\frac{V_{i}}{\sigma_{g}^{2}}\right)}^{\alpha } \end{array}$$

**Algorithm 2** Homeostatic Unsupervised Learning of Kernels: $\Phi =H(y;\eta ,\eta_{h},N_{0})$
1:Initialize the point nonlinear gain functions $z_{i}$ to similar cumulative distribution functions,2:Initialize *N* atoms $\Phi_{i}$ to random points on the *M*-unit sphere,3:**for***T* epochs **do**:4: draw a new batch y from the database of natural images,5: **for** each data point $y_{k}$ **do**:6:  compute the sparse representation vector using sparse coding $a_{k}=S\left(y_{k};\Psi =\left\{\Phi ,z,N_{0}\right\}\right)$,7:  modify atoms: $\forall i,\Phi_{i}\leftarrow \Phi_{i}+\eta \;\cdot \;a_{k,i}\;\cdot \;\left(y_{k}-\Phi a_{k}\right)$,8:  normalize atoms: $\forall i,\Phi_{i}\leftarrow \Phi_{i}/\left|\;\right|\Phi_{i}\left|\;\right|$,9:   update homeostasis functions: $\forall i,z_{i}\left(\;\cdot \;\right)\leftarrow \left(1-\eta_{h}\right)\;\cdot \;z_{i}\left(\;\cdot \;\right)+\eta_{h}\;\cdot \;\delta \left(a_{k,i}\leq \;\cdot \;\right)$.


This is similar to the mechanisms of gain normalization proposed by the authors of [14], which were recently shown to provide efficient coding mechanisms by the authors of [42]. However, compared to these methods which manipulate the gain of dictionaries based on the energy of coefficients, we propose to rather use a methodology based on the probability of activation. Indeed, the main distortion that occurs during learning is on higher statistical moments rather than variance, for instance when an atom is winning more frequently during the earliest iterations, its pdf will typically be more kurtotic than a filter that has learned less.

Recently, such an approach was proposed by the authors of [23]. Based on the same observations, the authors proposed to optimize the coding during learning by modulating the gain of each dictionary element based on the recent activation history. They base their Equalitarian Matching Pursuit (EMP) algorithm on a heuristics, which cancels the activation of any filter that was more often activated than a given threshold probability (parameterized by $1+\alpha_{h}$). In our setting, we may compute a similar algorithm using an evaluation of the probability of activation followed by binary gates

(9) $$\begin{array}{c} p_{i}\leftarrow \left(1-\eta_{h}\right)\;\cdot \;p_{i}+\eta_{h}\;\cdot \;1/K\sum_{k=1\cdots K}\delta \left(a_{i,k}>0\right) \end{array}$$

(10) $$\begin{array}{cc} \; & \mathrm{and}\gamma_{i}=\delta \left(p_{i}<N_{0}/N\;\cdot \;\left(1+\alpha_{h}\right)\right) \end{array}$$

As such, $p_{i}$ is an approximation of the average activation probability based on a moving average controlled by the learning parameter $\eta_{h}$. Interestingly, they reported that such a simple heuristic could improve the learning, deriving a similar result as we have shown in Figure 1 and Figure 2. Moreover they have shown that such a homeostatic mechanism is more important than optimizing the coding algorithm, for instance by using OMP instead of MP. Again, such strategy can be included in line 9 of the learning algorithm.

**Figure 3:**
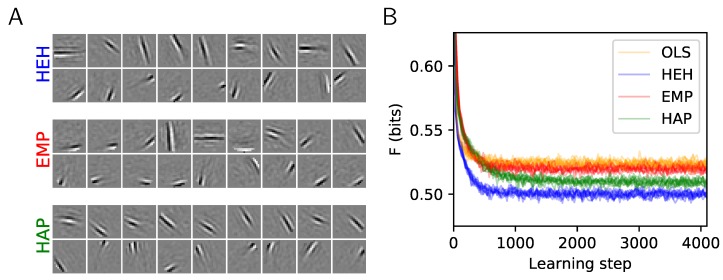
Homeostasis on Activation Probability (HAP) and a quantitative evaluation of homeostatic strategies. (**A**) The plot shows 18 from the N=676 dictionaries learned for the two heuristics EMP and HAP and compared to the optimal homeostasis (see Figure 1A, HEH). Again, the upper and lower row respectively show the least and most probably selected atoms. (**B**) Comparison of the cost *F* during learning and cross-validated over 10 runs: The convergence of OLS is similar to EMP. The simpler HAP heuristics gets closer to the more demanding HEH homeostatic rule, demonstrating that this heuristic is a good compromise for fast unsupervised learning.

Similarly, we may derive an approximate homeostasis algorithm based on the current activation probability, but using an optimization approach on the gain modulation. Ideally, this corresponds to finding $\gamma_{i}$ such that we minimize the entropy $-\sum_{i=1\cdots N}p_{i}\log p_{i}$. However, the sparse coding function $S(y_{k};\Psi =\left\{\Phi ,z,N_{0}\right\})$, which would allow to compute $p_{i}$ is not differentiable. A simpler approach is to compute the change of modulation gain that would be necessary to achieve an uniform probability. Indeed, such “equiprobability” is the known solution of the maximum entropy problem, that is when $\forall i,p_{i}=p_{0}\overset{def.}{=}N_{0}/N$:

(11) $$\begin{array}{c} p_{i}\leftarrow \left(1-\eta_{h}\right)\;\cdot \;p_{i}+\eta_{h}\;\cdot \;1/K\sum_{k=1\cdots K}\delta \left(a_{i,k}>0\right) \end{array}$$

(12) $$\begin{array}{cc} \; & \mathrm{and}\gamma_{i}=\frac{\log(1/p_{i})}{\log(1/p_{0})}=\frac{\log(p_{i})}{\log(p_{0})} \end{array}$$

where $\eta_{h}$ controls as above the speed of the sliding average for estimating the activation probability. Note that the gain is equal to one if the activation probability reaches the target probability. It becomes excitatory or inhibitory for cells whose probability is, respectively, below or above the target. Assuming an exponential probability distribution function for the sparse coefficients before the thresholding operation, this expression follows as the solution to scale coefficients such that overall each neuron fires with equal probability. We will coin this variant of the algorithm Homeostasis on Activation Probability (HAP). Following these derivations, we quantitatively compared OLS, EMP, and HAP to HEH (see Figure 3). This shows that although EMP slightly outperforms OLS (which itself is more efficient than None, see Figure 2B), HAP proves to be closer to the optimal solution given by HEH. Moreover, we replicated in HAP the result of [23] that while homeostasis was essential in improving unsupervised learning, the coding algorithm (MP vs. OMP) mattered relatively little (see Annex (https://spikeai.github.io/HULK/#Testing-different-algorithms)). Also, we verified the dependence of this efficiency with respect to different hyperparameters (as we did in Figure 2B). Overall, these quantitative results show that the HEH algorithm could be replaced by a simpler and more rapid heuristic, HAP, which is based on activation probability. This would generate a similar efficiency for the coding of patches from natural images.

## 3. Discussion and Conclusions

One core advantage of sparse representations is the efficient coding of complex multidimensional signals such as images using compact codes. Inputs are thus represented as a combination of few elements drawn from a large dictionary of atoms. A common design for unsupervised learning rules relies on a gradient descent over a cost measuring representation quality with respect to sparseness. This constraint introduces a competition between atoms. In the context of the efficient processing of natural images, we proposed here that such strategies can be optimized by including a proper homeostatic regulation enforcing a fair competition between the elements of the dictionary. We implemented this rule by introducing a nonlinear gain normalization similar to what is observed in biological neural networks. We validated this theoretical insight by challenging this adaptive unsupervised learning algorithm with different heuristics for the homeostasis. Simulations show that at convergence, although the coding accuracy did not vary much, including homeostasis changed, qualitatively, the learned features. In particular, including homeostasis resulted in a more homogeneous set of orientation selective filters, which is closer to what is observed in the visual cortex of mammals [16,17,18]. To further validate these results, we quantitatively compared the efficiency of the different variants of the algorithms, both at the level of homeostasis (homeostatic learning rate, parameters of the heuristics), but also to the coding (by changing *M*, *N* or $N_{0}$) and to the learning (by changing the learning rate, the scheduling or *M*). This demonstrated that overall, this neuro-inspired homeostatic algorithm provided with the best compromise between efficiency and computational cost.

**Figure 4:**
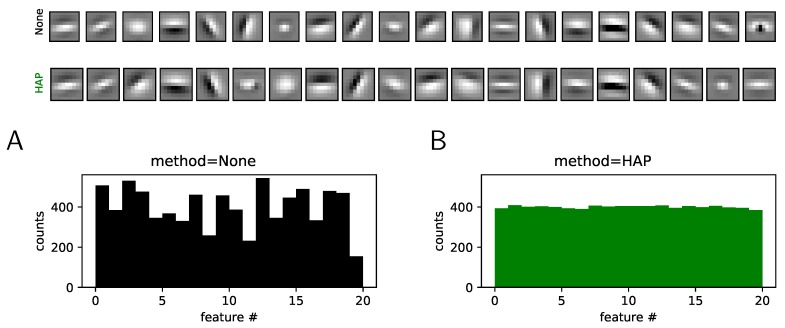
Extension to Convolutional Neural Networks (CNNs). We extend the HAP algorithm to a single-layered CNN with 20 kernels and using the ATT face database. We show here the kernels learned without (None, top row) and with (HAP, bottom row) homeostasis (note that we used the same initial conditions). As for the simpler case, we observe a heterogeneity of activation counts without homeostasis, that is, in the case which simply normalizes the energy of kernels (see (**A**)). With homeostasis, we observe the convergence of the activation probability for the different kernels (see (**B**)). This demonstrates that this heuristic extends well to a CNN architecture.

In summary, these results demonstrate that principles observed in biological neural computations can help improve real-life machine learning algorithms, in particular, for vision. Indeed, by developing this fast learning algorithm, we hope for its use in real-life machine learning algorithms. This type of architecture is economical, efficient and fast. The HAP algorithms uses only ReLUs such that it is easy to be transferred to most deep learning algorithms. Additionally, we hope that this new type of rapid unsupervised learning algorithm can provide a normative theory for the coding of information in low-level sensory processing, whether it is visual or auditory. Moreover, by its nature, this algorithm can easily be extended to convolutional networks such as those used in deep learning neural networks. This extension is possible by extending the filter dictionary by imposing the hypothesis of the invariance of synaptic patterns to spatial translations. Our results on different databases show the stable and rapid emergence of characteristic filters on these different bases (see Figure 4 and Annex (https://spikeai.github.io/HULK/#Testing-different-algorithms)). This result shows a probable prospect of extending this representation and for which we hope to obtain classification results superior to the algorithms existing in the state-of-the-art. As such, empirical evaluations of the proposed algorithms should be extended. For instance, it would be very useful to test for image classification results on standard benchmark datasets.

## Supplementary Materials

All scripts to reproduce figures in this paper are available at: https://spikeai.github.io/HULK. More information and pointers to the open-sourced code and supplementary control simulations are available at: https://laurentperrinet.github.io/publication/perrinet-19-hulk/.
